# Eye Care for Children in Special Schools: An Audit of Provision

**DOI:** 10.22599/bioj.166

**Published:** 2021-02-02

**Authors:** Louise C. Allen, Annette Dillon, Pamela Bowen

**Affiliations:** 1 University of Liverpool, GB; 2 Manchester Local Care Organisation, GB; 3 Wrightington, Wigan and Leigh NHS Foundation Trust, GB

**Keywords:** Special School, SEN, Special Educational Needs, Learning Disability

## Abstract

**Introduction:**

Children and young people with Special Educational Needs (SEN) are 28 times more likely to have eye problems than their typically developing peers. An ideal approach to the eye care for children attending special schools in England has been developed. Work in this area continues to evolve; therefore, an audit about existing services across the United Kingdom (UK) was undertaken.

**Method:**

A survey to ascertain key aspects of services for children with SEN that exist in the UK was developed and disseminated via Survey Monkey and at British and Irish Orthoptic Society (BIOS) events to all leads of the orthoptic profession.

**Results:**

Ninety-four service areas replied to the survey. Of these, 65 areas provide a special school service, 30 also provide a specialist service for SEN’s in hospital/community clinics; five provide only a specialist service in hospital/community clinics, and 24 reported no specialist service provision, outside that provided to everyone. In the school environment, 29 (44%) areas include vision and orthoptic assessment, whereas 31 (48%) include vision, orthoptic, and refraction assessment. All but two services were reported as orthoptic-led, 26 (40%) special school services involved optometric input within school, and no services had optical dispensing within school.

**Discussion:**

The results of this survey suggest that access to all aspects of eye care is not always available in school where a service exists. Families have to travel to the hospital or community optometrist for further assessment, which is not suitable in a number of cases, though it may be desirable, in some.

## INTRODUCTION

Children with Special Educational Needs (SEN) are estimated 28 times more likely (SeeAbility 2016; Emerson and Robertson 2011) to have an eye problem than their typically developing peers. Nielson, Skov, and Jenson (2007) report prevalence of myopia of to be 10.8%, hypermetropia, 15.3%, astigmatism, 20.6%, and strabismus 26.8% in children with learning disability (LD). A similar study found that a new glasses prescription would be beneficial for up to 26.6% (N = 172) of children assessed in special school (Das et al. 2010). This increased risk of visual problems in children with SEN results from a variety of reasons related to the underlying aetiology for their learning disability. Currently, there is no gold standard for surveillance of vision/eyes for children with SEN; however, recent findings have shown that the standard National Screening Committee (NSC) recommended vision screening is not suitable for this group of children (Donaldson et al 2019; Public Health England 2019; Hall and Elliman 2006). The NHS Long Term Plan (2019) has committed to bring hearing, sight, and dental checks to all children and young people attending residential special schools; this extends to all special school placements. Therefore, the impetus and potential funding is now available in England to improve access to eye care for this high-risk group.

Several researchers have undertaken studies to ascertain the ideal standard of provision and identify unmet need (Black et al. 2019; Donaldson et al. 2019; Woodhouse et al. 2014). The Framework for Provision of Eye Care in Special Schools in England (2016) provides a consensus on the expected standard.

A lack of equality for children with SEN has been identified. Work carried out in England by the charity, SeeAbility quote that 44% (n = 1500) of children attending special school have never had eye care before (SeeAbility 2019). Similar work in Wales quotes that 42.2% (n = 173) of their study group had never had a previous eye test (Woodhouse et al. 2014). These findings, however, were based on parental memory of previous care and work of this kind has been targeted specifically for areas that lack funded services within special school. These findings may be lower in other areas of the UK, but this data is unavailable. The reason for undertaking this audit was to identify what eye care services exist, in the UK, for children with SEN, and although the orthoptic profession has previously completed audits of provision, there remains no published record of what services currently exist. Particularly the services from the orthoptic profession, and their substantial role within the multi-disciplinary team.

The reasons for a lack of data in this area are multifactorial. Classification of Special Educational Needs, Learning Disability, and/or Autism make identification of this population difficult. In addition to this, there is no national registration for people with learning disability and their health needs are extremely heterogeneous. SEN’s are associated with a general diagnosis, of which there are numerous, including varying degree. Much of the literature around assessment is based on participants with typical development. Despite this, evaluation of services in order to reduce avoidable sight loss in children who may not otherwise express or demonstrate obvious signs and symptoms is required.

The aim of this audit was to seek information about orthoptic/optometric/ophthalmic services for children with SEN around the UK. This was undertaken to summarise the areas in the UK where provision of eye care for children with SEN are currently available, and to identify where they are not. This is pertinent because current work undertaken by the government, in England, and work undertaken in Wales and Ireland aims to significantly improve access to eye care for children with SEN and LD (Woodhouse et al. 2012; SeeAbility 2019; Black et al. 2019). A baseline of service provision would therefore be considered useful for future enhancement.

## METHODS

The BIOS has a clinical advisory group (CAG) for Orthoptics related to SEN. The steering group of the CAG designed a six-question survey to address the aims set out by the audit. Questions were designed to ascertain information about where the services took place, the professionals that were involved, and the type of assessments offered within special school. The number of questions were kept to a minimum to allow adequate response rates to the survey. The questions were refined by the CAG steering group and sent for approval to the BIOS. The term vision assessment has been interpreted as visual acuity alone; this is because any further assessments of vision such as findings from a functional vision assessment are usually considered part of the orthoptic assessment. A pilot response taken by an orthoptic department addressed the need to include a section for comments. The survey was deemed appropriate to determine the key aspects about service delivery in different regions of the UK. The survey was implemented via Survey Monkey, in one email and a follow-up reminder. It was sent to all heads and leads of the orthoptic profession registered with the BIOS. Printed copies of the survey were also produced and handed out at two BIOS events. One of these events was the SEN CAG study day and the other, the BIOS Leads of the Orthoptic Profession meeting; both were national events held in Liverpool and Birmingham. The regions of the UK were categorised using the same numerical system as previously published by Mazzone, Carlton and Griffiths (2017) in their BIOS Vision Screening Audit.

Ethical approvals for this survey were not required because no data were collected that reach beyond that gained through standard patient care. Permission to share the information was sought in a statement introducing the survey, explaining that a report would be published detailing the key aspects of service delivery for children with SEN.

## RESULTS

Responses for 94 areas of the UK were received; these areas may serve approximately one to six special schools. The number of special schools per area was not collected. However, individual members of the BIOS SEN clinical advisory group report provision of 1–6 special schools reflects the approximate size of their catchment area. While the survey was sent to all areas of the UK, responses were received only from Wales, England, and Northern Ireland.

**Figure 1:**
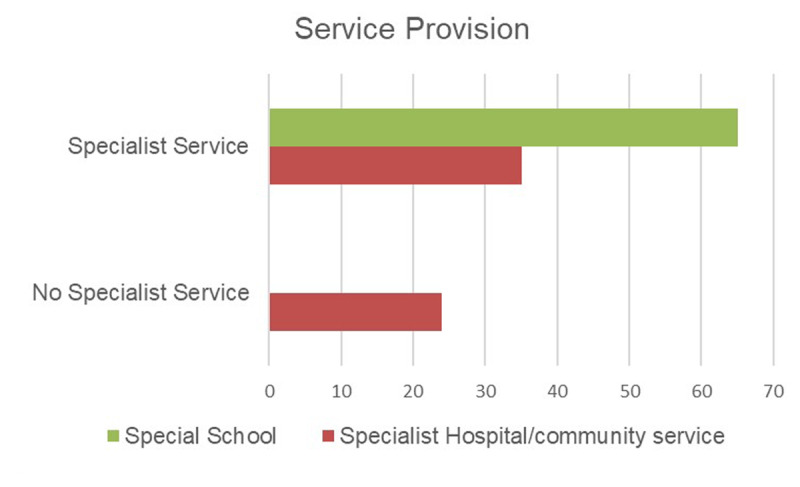
Graph to show number of services reported across the UK.

The survey asked whether the department provided a specialist service for children within special schools, and whether a specialist service was provided elsewhere (i.e., within hospital or community clinic). Within the 94 replies, the majority (65 areas) provided a special school service, 35 areas reported provision of a specialist SEN/LD service within hospital or community-based centres such as children’s centres, and 30 areas provided specialist services both within and out of special school. Twenty-four sites reported that no specialist service existed in their area, with supplementary comments to explain that their services integrated provision for all patients within their hospital/community clinics (***Figure 1***).

**Figure 2:**
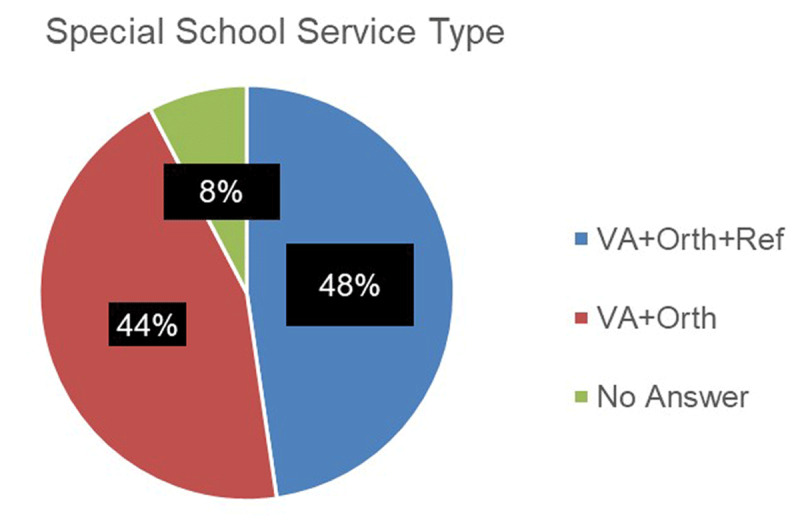
Chart to show the type of special school service provided (VA = Visual Acuity; Orth = Orthoptic Assessment; Ref = Refraction).

The survey also asked ‘what type of special school service was being provided’ The question asked whether the service provided vision assessment only, vision plus orthoptic assessment, or vision, orthoptic assessment, and refraction within school. It is recognised that there are a variety of approaches adopted dependent on local resources, requirements, and historical pathways of delivery. ***Figure 2*** Shows that 48% include a refraction in school whereas 44% do not. Of those services providing refraction, 8 (26%) were provided by an ophthalmologist, whereas 23 (74%) were provided by an optometrist. No services were reported to provide glasses dispensing within the school environment.

Services were orthoptic-led, except two. One was reported to be led by the optometrist and another led by the ophthalmologist. ***Table 1*** shows the number of eye professionals involved in the services provided within special school.

**Table 1:** Showing Eye Professionals Involved within the Reported Services.


TEAM MEMBER	NUMBER

**Ophthalmologist**	13

**Optometrist**	26

**Orthoptist**	65


*Note*: Other professionals are involved in Orthoptic-led SEN services, such as the Qualified Teacher of the Visually Impaired, wider multi-disciplinary team such as the physiotherapist, paediatrician, teachers, and nurses; however, these professionals are provided and governed by external departments, thus not included in this audit.

## DISCUSSION

The results confirm that there remains a significant unmet need. There are approximately 120,000 children attending specials schools in England (SeeAbility 2019) and the services described herein would only account for approximately 32,000 children across the UK. In addition to this, there is a variety of service provisions for those children living in an area fortunate to have access to a special school service. For children in 24 areas, no specialist service exists, whereas in 65 areas they can access eye care within the school environment. Within the school environment, only 31 (48%) can access a refraction. Services provided by SeeAbility also include the provision of glasses dispensing (Black et al. 2019; Donaldson et al. 2019). This is similar to other proposed schemes/projects such as the School Pupil Eye Care Service (SPECS) in Wales and Special Education Eyecare (SEE) Project in Northern Ireland (Welsh Government 2016; Black et al. 2019). Families are reported to take up the offer of in-school dispensing 99.6% of the time (SeeAbility 2019). Though this may be ideal for some localities, it may be less practical in others due to access to repairs out of school time or strong existing relationships with ophthalmic dispensers in the community. Relationships built over time with local community services may aid transition when the young person leaves school.

There are several limitations to this survey. While the electronic version of the survey was sent to all leads of the orthoptic profession, a hard copy version was only distributed at two events, both of which were held in England. The data described does not include services provided by others; SeeAbility is the only other known provider of such a service, and they have to date funded and provided over 3500 eye tests to 1500 children (SeeAbility 2019). This survey provides limited information about the services that do exist. Further work is required to ascertain information about activity. Individual local audit data is available but remains unpublished. A national audit to benchmark against key performance indicators for a gold standard service would be beneficial. The terminology used within the survey could have been interpreted in slightly different ways; for example, a refraction appointment in some services would typically include a fundus and media evaluation but not in others.

There may be reasons for inequality and lack of service provision, which prove challenging to address. The results show that around half of the services within special school involved a refraction, usually undertaken by an optometrist. Children are required to visit a community clinic or hospital for their refraction in the other half of cases. All services involved an orthoptic assessment, which includes an extensive assessment of visual function and functional vision. This type of assessment, particularly in children with SEN, is required more often during their developmental years, and changes in response to management strategies. As a refraction is required less frequently than the orthoptic assessment in most cases, many services are set up with orthoptic involvement only. It may be more difficult to ascertain optometric services within school due to the limited number of optometrists with an interest in this area, whereas orthoptists are the experts in assessment of children’s vision (Royal College of Ophthalmologists 2015) at the outset of their training (BIOS 2016). Occasionally, the ophthalmologist is reported to provide care within special school. The limited number of ophthalmologists attending special school could be due to the shortage of paediatric ophthalmologists in some areas and the tariff included for their time (Framework for Eye Care in Special Schools in England 2016). The lack of dispensing opticians within any services described here is due to the limited number of Hospital Eye Services working directly with a dispenser. Parents are typically directed to the community optometric practice to obtain their glasses from a provider of their choice. There are also examples of individual service designs where vision support groups and Qualified Teachers of the Visually Impaired (QTVI) are involved with the special school eye care provision to various degrees. Anecdotal evidence also supports models of care whereby services are provided to an optimal degree by a combination of in-school, hospital, and community optometry practices.

## CONCLUSION/RECOMMENDATIONS

Ideally, children in special school benefit from a comprehensive assessment provided by a multidisciplinary (MDT) eye care team with flexibility in the approach to allow for local resource, environment, and service-user preference. The multi-disciplinary team requires input from orthoptists, optometrists, ophthalmologists, and dispensing opticians.

A specialist assessment is required, undertaken with an orthoptist at aged 4–5years (NSC 2018; Hall 2006) and subsequent follow-up by the MDT eye care team, should a problem be identified. There remains limited evidence that continuing to provide yearly visual assessments after 4–5 years is cost effective in the typically developing population (Salebo & Rahi 2013). However, due to the variability in assessment responses, tests used, and higher risk of visual problems (Black et al. 2019; Donaldson et al. 2019) a yearly visual assessment, including all aspects of visual functioning and eye movements, which may or may not involve a refraction fundus and media evaluation, is recommended for children with SEN who attend special school.

All but two of the services described are orthoptic-led, perhaps because the orthoptist is often a known member of the multi-disciplinary team at a Trust and is well placed to work inter-professionally. The main barrier to inter-professional working within this area is the different sources of funding provided from each area of their care. Sustainability and transformation partnerships where system-wide goals provided ultimately by an Integrated Care System could be a solution to this, avoiding duplication and omission of care.
